# Supplementary material for: Collagenase Activity of Bromelain Immobilized at Gold Nanoparticle Interfaces for Therapeutic Applications
Source: Pharmaceutics. 2021 Jul 27;13(8):1143. doi: 10.3390/pharmaceutics13081143 (PMC8401344; doi:10.3390/pharmaceutics13081143)
Supplement: Supplementary file 1 [file pharmaceutics-13-01143-s001.zip › pharmaceutics-1259027-supplementary.pdf]

pharmaceutics

MDPI

Supplemental Materials: Collagenase Activity of Bromelain Immobilized at Gold Nanoparticle Interfaces for Therapeutic Applications

Adrianne M. M. Brito, Vitor Oliveira, Marcelo Y. Icimoto * and Iseli L. Nantes-Cardoso *

1. Supplemental Material

1.1. Confocal Laser Scanning Microscopy (CLSM) image-based analysis

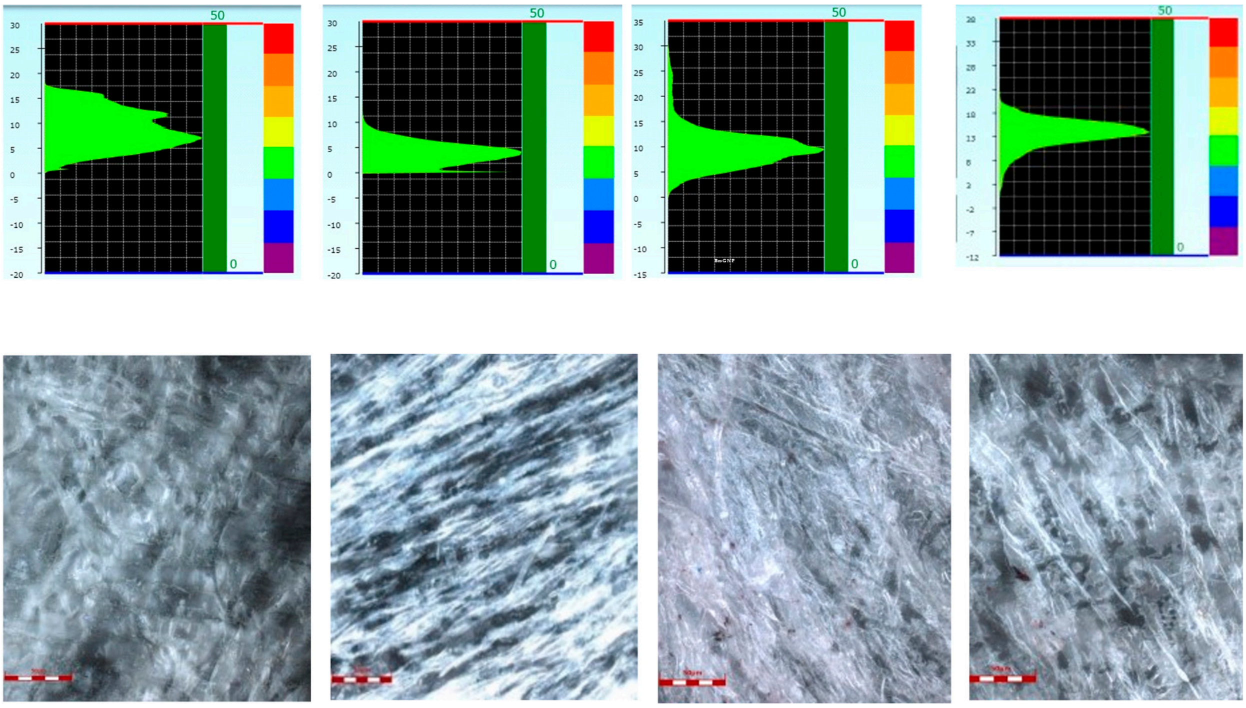

Figure S1. Confocal Laser Scanning Microscopy (CLSM) image-based analysis showing the roughness distribution profile (upper) acquired of the analysis of the respective optical scanning image (500×) (lower). From left to right collagen membrane incubated with HEPES buffer, with papain; with NanoBro and NanoBro in the presence of E-64.

CLSM is a technique that has been widely used in Material Science to characterize surface roughness of various materials after undergoing degradation treatments or processes [1–3]. It has also been employed to obtain in-depth and high-resolution images of complex biological tissues[1–3] due to the ability to selectively collect information from a specific plane and then to group it for three-dimensional reconstruction of the topography. As different focal planes can be chosen, it is possible to produce Z-stacks by 3D scanning of horizontal planes and in this case, presenting the overlapping planes from the surface to a maximum chosen depth. Thus, in the present study, this technique was employed with the objective of, from this image, construct the roughness profile of the collagen membrane, quantifying the surface roughness through several parameters. The contrast in the images is a result of differences in the refractive index of materials, which, although typically small in biological tissues, becomes very useful after working on equipment-specific software. So, because this technique enables the elimination of out-of-focus information from the image, it favors the acquisition of images from thicker samples, such as bacterial biofilms and collagen membranes[1–3].

It is important to note that the width of the base of the curve representative of the roughness distribution profile is related to the heterogeneity of the roughness pattern in

relation to the peak height. Thus, the narrower and closer to the shape of a Gaussian curve, the smaller the depth difference between the roughness and, therefore, the membrane has a smoother appearance. The greater the displacement of this distribution profile towards the red of the lateral color scale, the greater the height of the peaks, just as the displacement towards the violet indicates a greater presence of deeper valleys. However, due to the complexity of the topography of the collagen membrane and the different selectivity in the activity of free enzymes and associated with nanostructures, the analysis performed is of the semi-quantitative type and related to the enzyme's mode of action and not the amount of activity.

References

1. USHARANI, N.; JAYAKUMAR, G.C.; RAO, J.R.; CHANDRASEKARAN, B.; NAIR, B.U. A microscopic evaluation of collagen-bilirubin interactions: *in vitro* surface phenomenon. *J. Microsc.* **2014**, *253*, 109–118, doi:10.1111/jmi.12101.
2. J. R. Soc. Interface 13: 20160088r, P.; Georgiadis, M.; Müller, R. Techniques to assess bone ultrastructure organization: orientation and arrangement of mineralized collagen fibrils., doi:10.1098/rsif.2016.0088.
3. Cao, S.; Li, H.; Li, K.; Lu, J.; Zhang, L. A dense and strong bonding collagen film for carbon/carbon composites. *Appl. Surf. Sci.* **2015**, *347*, 307–314, doi:10.1016/j.apsusc.2015.04.081.
